# GABA-A and GABA-B Receptors in Filial Imprinting Linked With Opening and Closing of the Sensitive Period in Domestic Chicks (*Gallus gallus domesticus*)

**DOI:** 10.3389/fphys.2018.01837

**Published:** 2018-12-19

**Authors:** Naoya Aoki, Shinji Yamaguchi, Toshiyuki Fujita, Chihiro Mori, Eiko Fujita, Toshiya Matsushima, Koichi J. Homma

**Affiliations:** ^1^Department of Life and Health Sciences, Faculty of Pharmaceutical Sciences, Teikyo University, Tokyo, Japan; ^2^Research Fellow of the Japan Society for the Promotion of Science, Tokyo, Japan; ^3^Graduate School of Arts and Sciences, The University of Tokyo, Tokyo, Japan; ^4^Department of Biology, Faculty of Science, Hokkaido University, Sapporo, Japan

**Keywords:** filial imprinting, sensitive period, GABA-A receptor, GABA-B receptor, thyroid hormone

## Abstract

Filial imprinting of domestic chicks has a well-defined sensitive (critical) period lasting in the laboratory from hatching to day 3. It is a typical model to investigate the molecular mechanisms underlying memory formation in early learning. We recently found that thyroid hormone 3,5,3′-triiodothyronine (T_3_) is a determinant of the sensitive period. Rapid increases in cerebral T_3_ levels are induced by imprinting training, rendering chicks imprintable. Furthermore, the administration of exogenous T_3_ makes chicks imprintable on days 4 or 6 even after the sensitive period has ended. However, how T_3_ affects neural transmission to enable imprinting remains mostly unknown. In this study, we demonstrate opposing roles for gamma-aminobutyric acid (GABA)-A and GABA-B receptors in imprinting downstream of T_3_. Quantitative reverse transcription polymerase chain reaction and immunoblotting showed that the GABA-A receptor expression increases gradually from days 1 to 5, whereas the GABA-B receptor expression gradually decreases. We examined whether neurons in the intermediate medial mesopallium (IMM), the brain region responsible for imprinting, express both types of GABA receptors. Immunostaining showed that morphologically identified putative projection neurons express both GABA-A and GABA-B receptors, suggesting that those GABA receptors interact with each other in these cells to modulate the IMM outputs. The roles of GABA-A and GABA-B receptors were investigated using various agonists and antagonists. Our results show that GABA-B receptor antagonists suppressed imprinting on day 1, while its agonists made day 4 chicks imprintable without administration of exogenous T_3_. By contrast, GABA-A receptor agonists suppressed imprinting on day 1, while its antagonists induced imprintability on day 4 without exogenous T_3_. Furthermore, both GABA-A receptor agonists and GABA-B receptor antagonists suppressed T_3_-induced imprintability on day 4 after the sensitive period has ended. Our data from these pharmacological experiments indicate that GABA-B receptors facilitate imprinting downstream of T_3_ by initiating the sensitive period, while the GABA-A receptor contributes to the termination of the sensitive period. In conclusion, we propose that opposing roles of GABA-A and GABA-B receptors in the brain during development determine the induction and termination of the sensitive period.

## Introduction

Newly hatched chicks undergo filial imprinting, a process in which they memorize and follow their mother in order to receive care (Lorenz, 1937; Vallortigara and Versace, 2018). The domestic chick (*Gallus gallus domesticus*) serves as a useful model for early learning and memory (Rose, 2000; Matsushima et al., 2003; Horn, 2004; Vallortigara, 2012a,b; Versace et al., 2018). Imprinting has clearly a sensitive or critical period after which chicks cannot be imprinted (Hess, 1959). The molecular mechanisms of memory formation in imprinting have been investigated intensively (Horn, 2004; Yamaguchi et al., 2008a,b, 2010, 2011; Solomonia and McCabe, 2015). We previously revealed that the thyroid hormone 3,5,3′ -triiodothyronine (T_3_) functions as a starter and recoverer of the sensitive period (Yamaguchi et al., 2012). After hatching, imprinting training induces rapid inflow of T_3_ into the brain, which makes the chicks imprintable. Intravenous T_3_ injection into the intermediate medial mesopallium (IMM), a critical brain area for imprinting acquisition (McCabe et al., 1981), makes chicks imprintable even after the sensitive period has closed. For instance, chicks injected with T_3_ on day 1 can also be imprinted on days 4–8. We call these phenomena induced by T_3_ injection “memory priming” (MP). Downstream of T_3_, the protein Wnt-2b is involved in the memory formation of imprinting (Yamaguchi et al., 2018). Pena and colleagues recently showed that T_3_ activates the mechanistic target of rapamycin (mTOR), which has been implicated in long-term potentiation (LTP) and long-term memory, in the IMM neurons and that mTOR activation by an Akt activator made day 4 chicks imprintable similar to the described T_3_ effects (Batista et al., 2018). However, the roles of neurotransmitters underlying the neuronal mechanisms downstream of T_3_ remain unknown. In a previous study, the exogenous injection of transmitters or hormones, e.g., norepinephrine, serotonin, dopamine, and testosterone, did not influence the imprintability of chicks, suggesting that they cannot be substituted for T_3_ (Yamaguchi et al., 2012).

Because mTOR signaling impairs GABAergic transmission (Weston et al., 2012), gamma-aminobutyric acid (GABA) is a candidate for neurotransmitters involved in T_3_ signaling during imprinting. Two types of GABA receptors, the ionotropic GABA-A receptor and the metabotropic GABA-B receptor, have different properties (Matsumoto, 1989) and are thought to be involved in memory processes (Venault et al., 1986; Chapouthier and Venault, 2002; Heaney and Kinney, 2016). In humans, an anterograde amnesia is caused by administration of the GABA-A receptor modulator diazepam (Lister, 1985; Mejo, 1992). In mice, learning is impaired after enhanced GABA-A signaling by diazepam. By contrast, the reduction of GABA-A signaling by an inverse agonist (methyl beta-carboline-3-carboxylate) enhances the memory processes in learning tasks. In the juvenile brains of mammals, the neural network development relies on the appropriate modulation of GABAergic neurons (Wu and Sun, 2015). In chicks, inhibitory GABAergic neurons are likely to be involved in filial imprinting. For example, the intraperitoneal injection of the GABA-A receptor modulator diazepam reduces the preference to the imprinting object (Venault et al., 1986). After 2 h of training, expression of the immediate-early gene *Fos* is increased in *Fos*-positive GABA-containing neurons of the IMM (Ambalavanar et al., 1999). In brain slices containing the left IMM, the GABA release in the presence of potassium is positively correlated with the preference score after 2 h of training (McCabe et al., 2001).

Therefore, we hypothesized that GABA-A and GABA-B receptors play a role as key determinants of the sensitive period for imprinting downstream of T_3_. We predicted that the expression levels of these two types of GABA receptors change around the sensitive period and that the balance between the two receptor types contributes to the beginning and the termination of the sensitive period. In this study, we determined the levels of GABA-A and GABA-B receptors and examined whether the GABA receptors are involved in imprinting using various GABA receptor agonists and antagonists. We found through these pharmacological experiments that GABA-B receptor signaling is necessary for imprinting, while GABA-A receptor signaling suppresses imprinting acquisition. We propose that the GABA-A and GABA-B receptor balance during development influences the start and end time points of the sensitive period.

## Materials and Methods

### Animals

The experiments were conducted under the guidelines of the national regulations for animal welfare in Japan and with the approval of the committee on animal experiments of Teikyo University (approval number: 12-019). In this study, 415 newly hatched domestic chicks of the Cobb strain (*G. gallus domesticus*) were used. Fertilized eggs were obtained from a local supplier (3-M, Aichi, Japan) and incubated at 37°C for 21 days. After hatching, the chicks were placed in dark plastic enclosures in a breeder at 30°C to prevent light exposure (Izawa et al., 2001).

### Gene Expression Analysis Using Quantitative Reverse Transcription Polymerase Chain Reaction

Quantitative reverse transcription polymerase chain reaction (RT-PCR) was performed as reported previously (Yamaguchi et al., 2008a; Takemura et al., 2018). Briefly, the telencephalons of 1-, 3-, and 5-day-old chicks reared in the dark were dissected under anesthetizing them using a ketamine (Daiichi Sankyo, Tokyo, Japan)-xylazine (Sigma-Aldrich Co., St. Louis, MO, United States) cocktail. Total RNA was extracted with TRIzol (Invitrogen, Carlsbad, CA, United States). Total RNA (1 μg) was treated with RNase-free DNaseI (Invitrogen) and used for quantitative RT-PCR. The relative expression levels were normalized to glyceraldehyde 3-phosphate dehydrogenase (GAPDH). The primers used were as follows: *GABA-A receptor subunit alpha 1* (XM_025154781), 5′-TGGGCTGGCAACCATTG -3′ (sense) and 5′- GCTTTGTTTCTGGCTTAACTTCTTTG -3′ (antisense); *GABA-B receptor subunit 2* (XM_015282399), 5′-TGACAATTTGGCTTGGGATTG -3′ (sense) and 5′-GGCTAAGAAACAACCAAATAACATCA -3′ (antisense); and *GAPDH* (XM_204305), 5′-TGGAGCCCCTGCTCTTCA-3′ (sense) and 5′-GGAACAGAACTGGCCTCTCACT-3′ (antisense).

### Immunoblot Analysis

An immunoblot analysis was performed as described previously (Yamaguchi et al., 2007). In brief, the telencephalons from 0- and 5-day-old chicks reared in the dark were dissected after anesthesia. For the detection of GABA-A receptors, an anti-GABA-A receptor subunit alpha 1 rabbit polyclonal antibody was used as the primary antibody (ab33299, 1:1,500; Abcam plc, Cambridge, United Kingdom), while an anti-rabbit horseradish peroxidase-conjugated antibody (1:1,000; GE Healthcare, Chicago, IL, United States) was used as the secondary antibody. To detect GABA-B receptors, an anti-GABA-B receptor subunit 2 rabbit monoclonal antibody (ab75838, 1:1,500; Abcam plc) was used, while an anti-rabbit horseradish peroxidase-conjugated antibody (1:1,000, GE Healthcare) was used as the secondary antibody. Data of each sample were normalized to the expression of beta-actin as detected by an anti-beta-actin mouse monoclonal antibody (A5316, 1:1,000, Sigma-Aldrich Co.). The band intensities were quantified using ImageJ (National Institutes of Health, Bethesda, MD, United States), and the ratios of the band intensities were calculated.

### Immunohistochemistry

Chicks on day 0 were transcardially perfused with 4% paraformaldehyde in phosphate buffered saline (PBS) under deep anesthesia using a ketamine-xylazine cocktail. The brains were post-fixed with the same fixative for 24 h and immersed in 30% sucrose in PBS. The brain tissues were then cut into 18-μm-thick sections using a cryostat. For fluorescent staining, the sections including the IMM (Kuenzel and Masson, 1988) were blocked with 3% normal pig serum for 1 h and incubated with anti-GABA-A receptor subunit alpha 1 goat polyclonal antibody (sc-31403, 1:250; Santa Cruz Biotechnology, Santa Cruz, CA, United States) and anti-GABA-B receptor subunit 2 rabbit monoclonal antibody (ab75838, 1:250; Abcam plc) for 24 h at 4°C. The sections were then incubated with Alexa Fluor 546-conjugated anti-goat antibody (1:250; Thermo Fisher Scientific K.K., Waltham, MA, United States), Alexa Fluor 488-conjugated anti-rabbit antibody (1:250; Thermo Fisher Scientific K.K.), and Hoechst 33342 (Thermo Fisher Scientific K.K.). Fluorescent images were obtained using a confocal microscope (FV-10i; Olympus, Tokyo, Japan).

### *In vivo* Injection

The injection was performed as described previously (Yamaguchi et al., 2011) with modifications. Chicks were anesthetized with a 1% isoflurane/air mixture and mounted on a stereotaxic apparatus. The skin was cut, and a small piece of the skull’s surface was incised. The dura mater was cut to expose the telencephalon. Stereotaxic coordinates for the IMM were as follows: 2.9 mm anterior to the bregma, 1.3 mm lateral to the midline, and 2.3 mm deep (Kuenzel and Masson, 1988). We slowly (13.4 nL/min) injected for 35 min GABA receptor drugs using an auto-nanoliter injector (Nanoject I; Drummond Scientific Co., Broomall, PA, United States). The GABA receptor drugs were GABA-A agonist: muscimol 5 mM (Wako Chemicals, Tokyo, Japan); GABA-A antagonist: bicuculline 5 mM (Wako), picrotoxin 5 mM (Wako); GABA-B agonist: baclofen 20 μM (Wako); GABA-B antagonist: CGP52432 1 mM (Tocris Bioscience, Bristol, United Kingdom); GABA 5 mM (Wako). The doses of the chemicals were determined with reference to Knudsen et al. (1993); Fedele et al. (1997); Campbell et al. (1999). Control chicks were subjected to a sham operation in which only the syringe was inserted into the IMM under anesthesia. The chicks were returned to the dark chamber at 30°C for 30 min to allow them to recover from the anesthesia. For the intravenous injection of baclofen, 200 μM baclofen was dissolved in PBS. For the intravenous injection of T_3_, 10 μM T_3_ (Sigma-Aldrich Co.) was dissolved in 0.002 M NaOH and 0.9% NaCl. In the experiment shown in Figure 4B, a low dose of bicuculline (0.33 mM) was injected into the IMM, and a low dose of baclofen (13.3 μM) was injected intravenously. The GABA receptor drugs used in each experiment are listed in Supplementary Table S1.

### Behavioral Training and Testing

Training for imprinting was performed according to the method of Izawa et al. (2001) with modifications. A hand-made training chamber (8 cm wide, 43 cm long, 15 cm high) was equipped with a rubber belt controlled by a microcomputer (RCX2.0; LEGO Co., Tokyo, Japan). Thirty minutes after the injection or the sham operation, two 1-h training sessions were conducted. An imprinting object (a blue LEGO block, 4.7 cm × 6.2 cm × 5.0 cm) was in one side of the training chamber. During training, the imprinting object rotated clockwise and anticlockwise repeatedly for 30 s with pauses of 10 s in between and was illuminated by a 100 W fiber optic light during the rotation. An infrared sensor was placed 20 cm in front of the imprinting object. If the chicks crossed the sensor, the belt moved toward the opposite side of the imprinting object, they did so again and again. We counted how many times the chicks crossed the infrared sensor during the training. The chick was not tested if the number was <500 for the sum of two training sessions. In our experiments, the injection of various chemicals did not impair the locomotor activities of the injected chicks. The locomotor activity was measured as previously described (Yamaguchi et al., 2012). In the simultaneous choice test, we used a T-maze with a 20-cm-long main arm and a 69-cm-long sidearm. The imprinting object (a blue LEGO block) and a novel control object (a brown LEGO block) were positioned at the end of each sidearm of the T-maze. After a chick started from the main arm, we counted the stay time of the approach area of each object during testing for 120 s. Except for the time the chicks stayed in the approach areas, they spent time in the intermediate area between two approach areas. We ran the tests four times and averaged the approach time. We then calculated a preference score by subtracting the approach time of the control object from the approach time of the imprinting object. After the behavioral experiments, the animals were sacrificed with an overdose of isoflurane.

### Statistical Analyses

For statistical analyses, we used R software for Windows (version 3.3.2; The R Foundation for Statistical Computing, Vienna, Austria) as previously described (Yamaguchi et al., 2018) or MATLAB for Windows (The Mathworks, Inc., Natick, MA, United States). Gene expression data are reported as mean ± standard error of the mean (SEM). All other data are presented as box plots. The number of animals used is indicated in each figure or legend. The equality of variance of each data point was checked by the *F*-test or Bartlett’s test. Since variances were not different in the quantitative RT-PCR data, we used the parametric two-way analysis of variance. Since variances were not different in the immunoblotting data, we used Student’s *t*-test. Since variances were significantly different in some data of the behavioral experiments, we used as a non-parametric test Steel’s multiple comparisons. *p*-values < 0.05 were considered significantly different. The *p*-values are shown in the Supplementary Table S2. We also determined Cohen’s *d* or η^2^ as effect size in the parametric analysis (Cohen, 1988). To determine the *r* value as the effect size for the non-parametric analysis, we calculated it from the *Z*-value of the Mann–Whitney *U* test according to the following formula: *r* = *Z*/√*n*. The effect sizes are shown in the Supplementary Table S3.

## Results

### Developmental Changes in the Gene Expression of GABA Receptors

**FIGURE 1:**
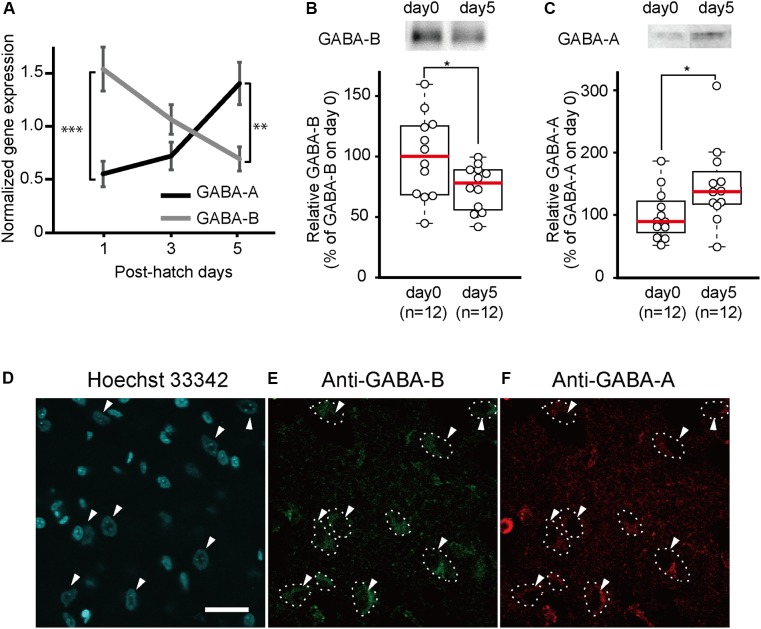
GABA-A receptor expression increases and GABA-B receptor expression decreases between day 1 and day 5. **(A)** Gene expression of GABA-A and GABA-B receptors in the telencephalons of 1-day-old (GABA-A: *n* = 8; GABA-B: *n* = 9), 3-day-old (GABA-A: *n* = 8; GABA-B: *n* = 9), and 5-day-old (GABA-A: *n* = 7; GABA-B: *n* = 7) chicks measured by quantitative RT-PCR. Black and gray indicates gene expression of GABA-A and GABA-B receptors, respectively. On day 1, the expression level of the GABA-B receptor is higher than that of the GABA-A receptor. The gene expression of the GABA-A receptor increases from days 1 to 5, whereas the gene expression of the GABA-B receptor decreases. On day 5, the GABA-A receptor expression level is higher than that of the GABA-B receptor. The gene expression was normalized to that of GAPDH [two-way analysis of variance; factor A: type of receptor; factor B: day, *F*_A__(1,42)_ = 2.52, n.s.; *F*_B__(2,42)_ = 0.64, n.s.; *F*_interaction__(2,42)_ = 14.42, *p* < 0.001; *F*_A_day1(1,42)_ = 19.15, ^∗∗∗^*p* < 0.001; *F*_A_day3(1,42)_ = 2.31, n.s.; *F*_A_day5(1,42)_ = 9.90, ^∗∗^*p* < 0.01; *F*_B_GABA-A(2,42)_ = 7.99, *p* < 0.01; *F*_B_GABA-B(2,42)_ = 7.07, *p* < 0.01]. **(B)** The protein expression levels of GABA-B receptors measured by immunoblotting. The expression of GABA-B receptors is presented as the percentage of the average GABA-B receptor expression on day 0. GABA-B receptors are significantly more expressed on day 0 than on day 5. (*t*-test, *t* = 2.18; ^∗^*p* < 0.05). **(C)** The protein expression of GABA-A receptors measured by immunoblotting. The expression levels of GABA-A receptors are shown as the percentage of the average GABA-A receptor expression on day 0. GABA-A receptor levels are significantly higher on day 5 compared to day 0. (*t*-test, *t* = 2.26, ^∗^*p* < 0.05). Images in **(B,C)** have been spliced together for illustrative purposes. The original data are shown in Supplementary Figure S1. **(D–F)** A sample was stained with Hoechst 33342 and immunostained with anti-GABA-A and anti-GABA-B antibodies. **(D)** The cell nuclei in the IMM are stained by Hoechst 33342. The arrowheads indicate the cell body of putative projection neurons. **(E)** The neurons in which GABA-B receptors are expressed are enclosed by dashed lines. **(F)** Dashed lines indicate neurons in which GABA-A receptors are expressed. GABA-A receptors are expressed in the same neurons as in **(E)**. Scale bar, 20 μm. n.s., not significant.

To measure the developmental changes in GABA-A and GABA-B receptor gene expressions after hatching, we conducted quantitative RT-PCRs. RNA was extracted from the brains of newborn chicks at days 1, 3, and 5. On day 1, the gene expression of the GABA-B receptor was significantly higher than that of the GABA-A receptor (Figure 1A). From days 1 to 5, the gene expression of the GABA-A receptor gradually increased, whereas that of the GABA-B receptor decreased. On day 5, the gene expression of the GABA-A receptor was significantly higher than that of the GABA-B receptor.

### Developmental Changes in the Protein Expression of GABA Receptors

To measure the developmental changes in GABA-A and GABA-B receptors in the telencephalon on days 0 and 5, we conducted immunoblotting using antibodies directed against GABA-A or GABA-B receptors. The amount of GABA-B receptors on day 0 in the telencephalon was significantly higher than that on day 5 (Figures 1B and Supplementary Figure S1A). In contrast, the amount of GABA-A receptors was significantly higher on day 5 than that on day 0 (Figures 1C and Supplementary Figure S1B). These results were consistent with the gene expression according to the quantitative RT-PCR experiments. This led us to the assumption that abundant GABA-B receptors on day 0 may facilitate imprinting at the start of the sensitive period while GABA-A receptors on day 5 suppress imprinting at the end of the sensitive period.

### Expression of GABA-A and GABA-B Receptors in IMM Neurons

Neurons in the IMM, a brain region responsible for imprinting acquisition, may express both GABA-A and GABA-B receptors and have opposing roles in imprinting. We conducted immunostaining using anti-GABA-A or anti-GABA-B antibody in brain slices containing the IMM region. Cell nuclei were stained by Hoechst 33342. Two types of cells in the IMM were distinguished based on their size (Figure 1D). Neurons with a cell body diameter > 15 μm were putatively projection neurons (Patel and Stewart, 1988; Tombol et al., 1988). These neurons in the IMM project to the arcopallium (Bradley et al., 1985) and intermediate hyperpallium apicale (IMHA) (Aoki et al., 2015). Among them, the pathway from the IMM to the IMHA plays critical roles in imprinting acquisition and recall (Aoki et al., 2015). As shown in Figures 1E,F, the majority of the larger neuronal cells expressed both GABA-A and GABA-B receptors, suggesting that the two receptor types may interact in the projection neurons to modulate the input from T_3_ in the IMM.

### Effects of GABA-B Receptor Agonists and Antagonists on Imprinting

**FIGURE 2:**
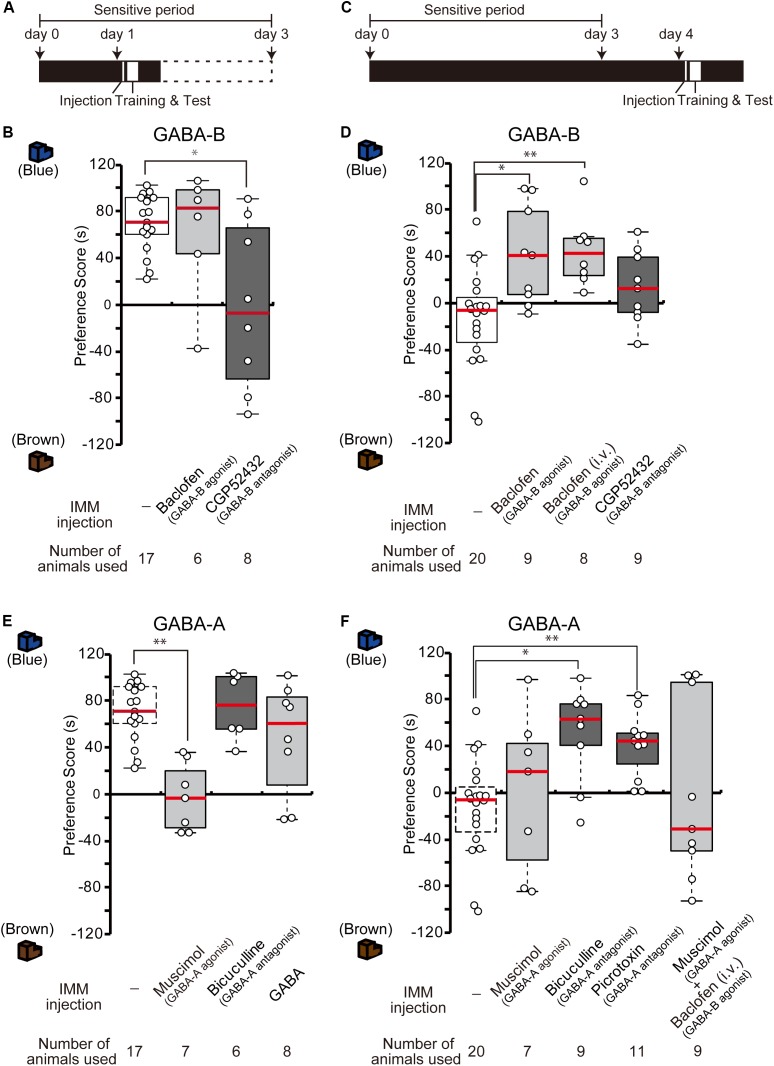
GABA-B receptors are necessary for imprinting, while GABA-A receptors suppress imprinting. **(A)** Schematic representation of the experimental schedule used in **(B,E)**. The chicks were bilaterally injected with drugs into the IMM right before training on day 1 during the sensitive period. **(B)** The chicks were injected with GABA-B receptor agonist or antagonist before training on day 1. The preference scores of the chicks injected with the GABA-B receptor antagonist CGP52432 are significantly lower than those of sham control chicks (Steel’s test, *t* = 2.38, ^∗^*p* < 0.05). The preference scores of chicks injected with the GABA-B receptor agonist baclofen are not significantly different from those of control chicks (Steel’s test, *t* = 0.56, n.s.). **(C)** Representation of the experimental design used in **(D,F)**. The chicks were injected with drugs into the IMM or intravenously right before the first training on day 4. **(D)** The chicks were injected with a GABA-B receptor agonist or antagonist before training on day 4. The preference scores of the chicks injected with the GABA-B receptor agonist baclofen into the IMM or intravenously were significantly higher than those of the control chicks (Steel’s test, IMM, *t* = 2.81, ^∗^*p* < 0.05; intravenously, *t* = 3.20, ^∗∗^*p* < 0.01). The preference scores of chicks injected with the GABA-B receptor antagonist CGP52432 do not differ from those of control chicks (Steel’s test, *t* = 1.58, n.s.). **(E)** The chicks were injected with a GABA-A receptor agonist or antagonist before training on day 1. The preference scores of the chicks that were injected with the GABA-A receptor agonist muscimol are significantly lower than those of the control chicks (Steel’s test, *t* = 3.53, ^∗∗^*p* < 0.01). The preference scores of the chicks injected with the GABA-A receptor antagonist bicuculline or GABA are not significantly different from those of the control chicks (Steel’s test, bicuculline, *t* = 0.56, n.s.; GABA, *t* = 0.81, n.s.). The sham control chicks’ data shown in **(B)** are duplicated for comparison. **(F)** The chicks were injected with a GABA-A receptor agonist or antagonist before training on day 4. The preference scores of the chicks injected with either the GABA-A receptor antagonist bicuculline or picrotoxin are significantly higher than those of the sham control chicks (Steel’s test, bicuculline, *t* = 2.96, ^∗^*p* < 0.05; picrotoxin, *t* = 3.61, ^∗∗^*p* < 0.01). The preference scores of chicks that were injected with the GABA-A receptor agonist muscimol are not different from those of the control chicks (Steel’s test, *t* = 0.66, n.s.). The preference scores of chicks that were injected with both the GABA-A agonist muscimol and the GABA-B agonist baclofen are not different from those of the control chicks (Steel’s test, *t* = 0.18, n.s.). The data of the control chicks shown in **(D)** are duplicated here for comparison. n.s., not significant.

The results from the GABA-B receptor expression experiment suggest that the abundant GABA-B receptors on day 1 may mainly mediate and facilitate imprinting. To examine whether the blockade of GABA-B receptors prevents day 1 chicks from imprinting, a GABA-B receptor antagonist (CGP52432) was injected into the IMM right before the training on day 1 (Figure 2A). The preference scores of chicks injected with the antagonist CGP52432 were significantly lower than those of the sham control chicks (Figure 2B). The preference scores of chicks injected with the agonist baclofen were not significantly different from those of the control chicks (Figure 2B). These results suggest that the molecular signaling of GABA-B receptor is necessary for imprinting acquisition on day 1.

We previously showed that the T_3_ levels in the brain decrease until day 4 after hatching, but that exogenous T_3_ injection extends the imprintable period even beyond the end of the sensitive period on day 4 (Yamaguchi et al., 2012). As the expression of GABA-B receptors also decreases until day 4, this decrease might be related to the end of the imprintable period. To examine whether the functional enhancement of GABA-B receptor makes day 4 chicks imprintable without an exogenous T_3_ administration, we injected the GABA-B receptor agonist baclofen into the IMM right before the training on day 4 (Figure 2C). The preference scores of the chicks injected with the agonist baclofen were significantly higher than those of the sham control chicks (Figure 2D). The intravenous injection of baclofen showed a similar effect on day 4 chicks (Figure 2D). These findings suggest that the GABA-B receptor agonist baclofen made day 4 chicks imprintable without exogenous T_3_ application. On the other hand, the preference scores of chicks injected with the antagonist CGP52432 were not significantly different from those of control chicks (Figure 2D). Taken together, these results suggest that the GABA-B receptor agonist baclofen substituted for the role of T_3_ and that GABA-B receptor signaling was downstream of T_3_.

### Effects of GABA-A Receptor Agonists and Antagonists on Imprinting

Due to the low expression of GABA-A receptors on day 1, these receptors may perform a different role than GABA-B receptors in the course of imprinting. To identify the role of GABA-A receptors, we injected the GABA-A receptor agonist muscimol into the IMM right before imprinting training on day 1 (Figure 2A). The preference scores of the chicks injected with the agonist muscimol were significantly lower than those of the sham control chicks (Figure 2E). By contrast, the preference scores of chicks injected with the GABA-A receptor antagonist bicuculline were not significantly different from those of control chicks (Figure 2E). These results suggest that an enhancement of GABA-A receptors suppresses imprinting processes. Furthermore, we injected GABA into the IMM right before imprinting training on day 1 (Figure 2A); however, the preference scores of the chicks injected with GABA were not significantly different from those of sham control chicks (Figure 2E). This result indicates that imprintability on day 1 does not depend on the GABA concentration but rather on the GABA-B receptor expression. GABA-A receptors might not have shown their suppressive role in imprinting due to their insufficient expression levels on day 1.

By contrast, the increased expression of GABA-A receptors on day 4 may prevent imprinting in chicks. Thus, we examined whether GABA-A receptor blockade would influence imprinting in chicks on day 4. The GABA-A receptor antagonists bicuculline or picrotoxin were injected into the IMM right before training on day 4 (Figure 2C). The preference scores of the chicks injected with these GABA-A receptor antagonists were higher than those of sham control chicks (Figure 2F). The preference scores of chicks injected with the GABA-A receptor agonist muscimol were not significantly different from those of control chicks (Figure 2F). These results demonstrate that a reduction in GABA-A receptor signaling made day 4 chicks imprintable without administration of T_3_ and that GABA-A receptor signaling was downstream of T_3_. When we injected both the GABA-A agonist muscimol and the GABA-B agonist baclofen at the same time, the chicks could not be imprinted (Figure 2F), probably because the ability of GABA-B receptors to accelerate imprinting was erased by the suppressive role of GABA-A receptors.

### Interaction Between the Roles of GABA-A and GABA-B Receptors in Imprinting

**FIGURE 3:**
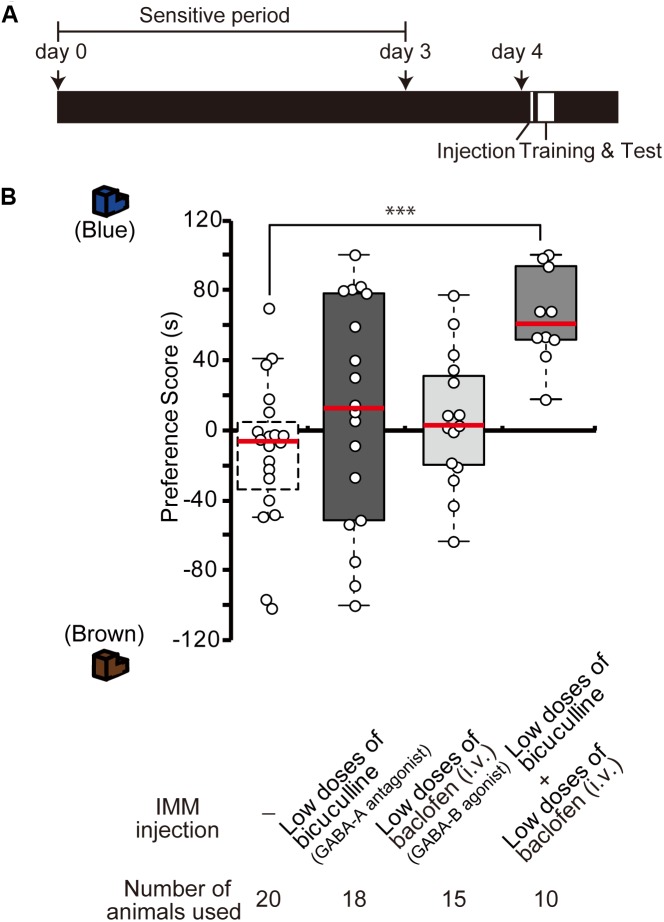
Interference of GABA-A and GABA-B signaling during imprinting. **(A)** Schematic representation of the experimental schedule used in **(B)**. The chicks were injected with drugs into the IMM or intravenously right before the first training on day 4. **(B)** The chicks were injected before training on day 4. The preference scores of the chicks injected with low doses of the GABA-A receptor antagonist bicuculline or low doses of the GABA-B receptor agonist baclofen are not different from those of control chicks (Steel’s test, bicuculline, *t* = 1.29, n.s.; baclofen, *t* = 1.37, n.s.). The preference scores of chicks that were injected with low doses of both chemicals are significantly higher than those of the control chicks (Steel’s test, *t* = 3.96, ^∗∗∗^*p* < 0.001). The data of sham control chicks shown in Figure 2D are duplicated for comparison. n.s., not significant.

To examine whether GABA-A receptor antagonist and GABA-B receptor agonist influence synergistically the imprinting in chicks, day 4 chicks were injected with low doses of the GABA-B agonist baclofen and the GABA-A antagonist bicuculline (Figure 3A). Either drug alone did not influence the imprinting (Figure 3B), but the combination of the two low-dose drugs clearly modulated the imprinting in chicks (Figure 3B). This indicates that GABA-A and GABA-B signaling interact synergistically with each other to enable imprinting.

### Effects of GABA Receptor Drugs on the Imprintability Induced by T_3_ Administration Before Training on Day 4

**FIGURE 4:**
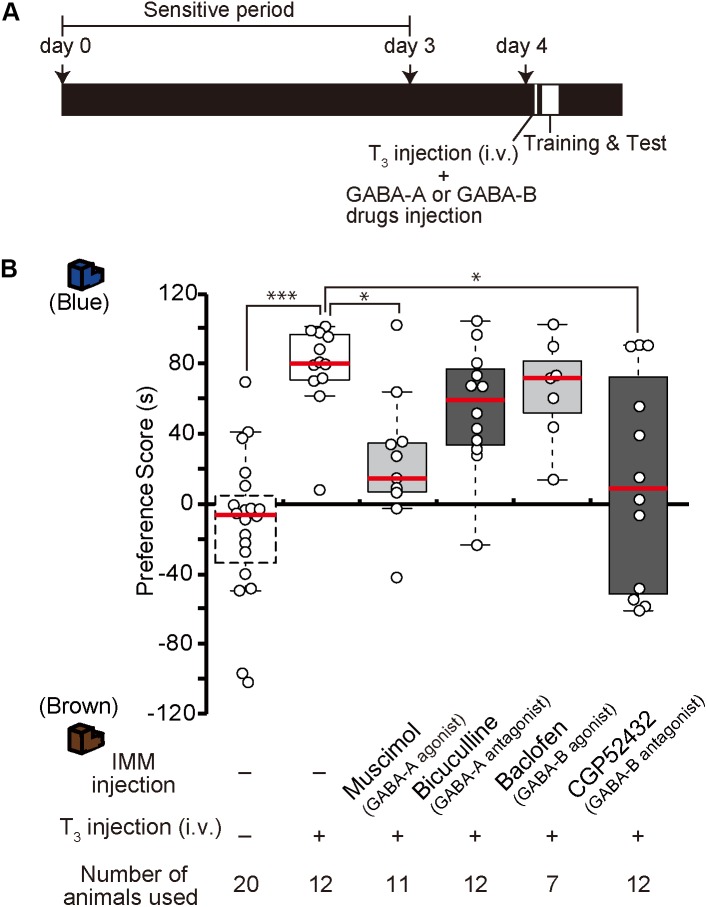
Enhancement of GABA-A signaling or reduction of GABA-B signaling on day 4 impairs the imprintability on day 4 induced by T_3_ injection on day 4. **(A)** The experimental schedule is shown schematically. GABA-A or GABA-B drugs were injected bilaterally into the IMM, and T_3_ was injected intravenously before the first training on day 4 after the sensitive period. **(B)** The preference scores of the chicks that were intravenously injected with T_3_ on day 1 are significantly higher than those of the control chicks (Steel’s test, *t* = 4.43, ^∗∗∗^*p* < 0.001). The preference scores of the chicks injected with the GABA-A receptor agonist muscimol and T_3_ are significantly lower than those of T_3_-injected chicks (Steel’s test, *t* = 2.89, ^∗^*p* < 0.05). The preference scores of the chicks injected with the GABA-B receptor antagonist CGP52432 and T_3_ are also significantly lower than those of T_3_-injected chicks (Steel’s test, *t* = 2.59, ^∗^*p* < 0.05). The preference scores of the chicks injected with both the GABA-A receptor antagonist bicuculline and T_3_ or both the GABA-B receptor agonist baclofen and T_3_ are not different from those of the T_3_-injected chicks (Steel’s test, bicuculline, *t* = 1.84, n.s.; baclofen, *t* = 0.92, n.s.). The data of the sham control chicks shown in Figure 2D are duplicated here for comparison. n.s., not significant.

In our previous study, we found that after T_3_ administration chicks are imprintable even beyond the sensitive period (Yamaguchi et al., 2012). To examine whether GABA-A receptor signaling is downstream of T_3_, the GABA-A receptor agonist muscimol was injected into the IMM, and T_3_ was injected intravenously right before training on day 4 (Figure 4A). The preference scores of chicks injected with both the GABA-A receptor agonist and T_3_ were significantly lower than those of control chicks injected with T_3_ alone (Figure 4B). This result means that GABA-A receptor signaling impaired the imprintability induced by T_3_ and was downstream of T_3_. To examine whether the GABA-B receptor signaling is also downstream of T_3_, the GABA-B receptor antagonist CGP52432 was injected into the IMM, while T_3_ was intravenously injected right before the training on day 4. The preference scores of chicks injected with both the GABA-B receptor antagonist and T_3_ were significantly lower than those of control chicks injected with T_3_ alone (Figure 4B). This result means that GABA-B receptor signaling downstream of T_3_ is necessary for acquiring imprinting. The preference scores of chicks injected with both the GABA-A receptor antagonist bicuculline and T_3_ or both the GABA-B receptor agonist baclofen and T_3_ were not different from those of T_3_-injected chicks.

### Effects of GABA Receptor Drugs Before T_3_ Injection on Day 1 to Induce MP

**FIGURE 5:**
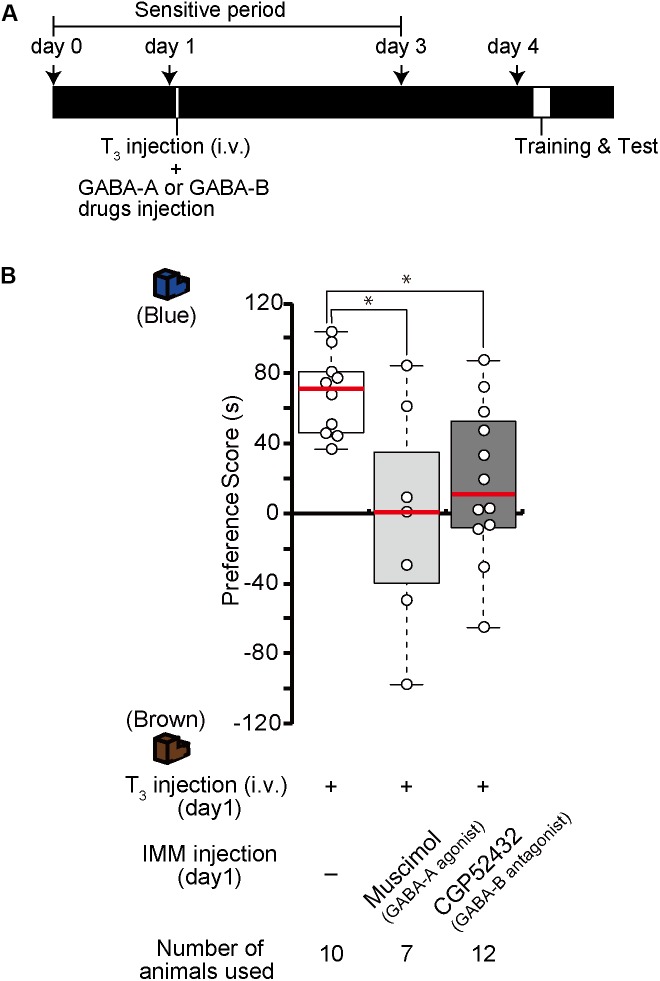
Enhanced GABA-A signaling or reduced GABA-B signaling on day 1 impairs memory priming on day 4 induced by T_3_ administration on day 1. **(A)** The experimental design is shown schematically. The chicks were injected with the GABA-A agonist muscimol or the GABA-B antagonist CGP52432 into the IMM and intravenously injected with T_3_ on day 1, then trained and tested on day 4. **(B)** The preference scores of the chicks that were injected with both T_3_ and the GABA-A receptor agonist muscimol are significantly lower than those of T_3_-injected chicks (Steel’s test, *t* = 2.24, ^∗^*p* < 0.05). The preference scores of the chicks that were injected with T_3_ and the GABA-B receptor antagonist CGP52432 are also significantly lower than those of T_3_-injected chicks (Steel’s test, *t* = 2.63, ^∗^*p* < 0.05).

The effects of one injection of exogenous T_3_ on imprinting were shown to last for more than 1 week (Yamaguchi et al., 2012). We call this phenomenon MP. To examine whether GABA-A receptor agonists or GABA-B receptor antagonists impair MP, we injected the GABA-A receptor agonist muscimol or the GABA-B receptor antagonist CGP52432 prior to the intravenous administration of T_3_ on day 1 (Figure 5A). The preference scores of these chicks were lower than those of the T_3_-injected control chicks (Figure 5B). These results indicate that GABA-A or GABA-B receptor signaling is involved at an earlier phase of MP.

### Effects of GABA Receptor Drugs on Day 4 Chicks Injected With T_3_ on Day 1

**FIGURE 6:**
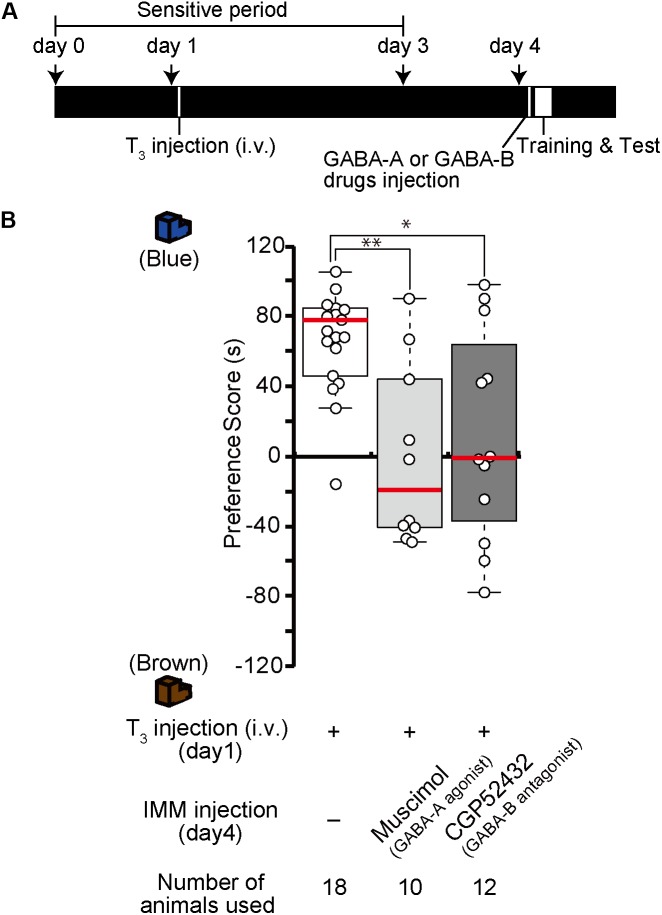
Enhancement of GABA-A signaling or reduction of GABA-B signaling on day 4 impairs memory priming on day 4 induced by T_3_ injection on day 1. **(A)** Schematic representation of the experimental design. The chicks were intravenously injected with T_3_ on day 1 and bilaterally injected with the GABA-A agonist muscimol or the GABA-B antagonist CGP52432 into the IMM shortly before a first training on day 4. **(B)** The preference scores of the chicks that were injected with T_3_ and the GABA-A receptor agonist muscimol are significantly lower than those of the T_3_-injected chicks (Steel’s test, *t* = 2.97, ^∗∗^*p* < 0.01). The preference scores of the chicks that were injected with T_3_ and the GABA-B receptor antagonist CGP52432 are also significantly lower than those of T_3_-injected chicks (Steel’s test, *t* = 2.24, ^∗^*p* < 0.05).

Because the effects of T_3_ injection on imprinting last for more than 1 week, structural and/or neural changes may occur in the IMM after T_3_ injection. To examine whether GABA-A receptor agonists or GABA-B receptor antagonists impair imprinting after such structural changes have already occurred in the brain, chicks were intravenously injected with T_3_ on day 1 and injected with the GABA-A receptor agonist muscimol or the GABA-B receptor antagonist CGP52432 into the IMM right before the training on day 4 (Figure 6A). The preference scores of these chicks were lower than those of T_3_-injected control chicks (Figure 6B). These results suggest that GABA-A or GABA-B receptor signaling is involved at a later stage of MP execution just before the imprinting training in addition to its role at the earlier step of MP described above.

### GABA Receptors as MP Executor in Imprinting

**FIGURE 7:**
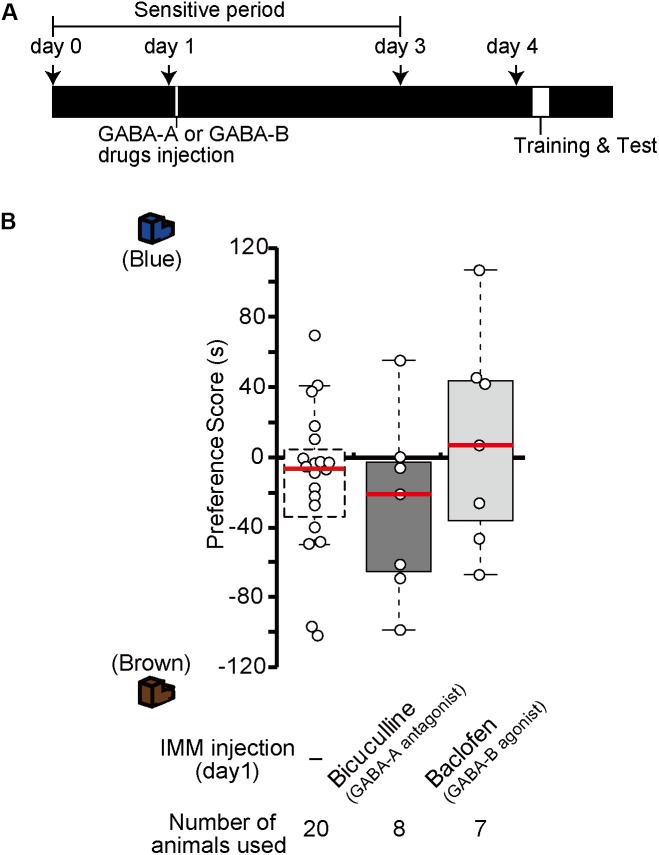
In the absence of exogenous T_3_, GABA-A receptor antagonists or GABA-B receptor agonists do not induce memory priming. **(A)** The schedule of the experiment. The chicks were injected with the GABA-A antagonist bicuculline or the GABA-B agonist baclofen into the IMM on day 1 and trained and tested on day 4. **(B)** The preference scores of the chicks injected with the GABA-A antagonist bicuculline or the GABA-B agonist baclofen are not different from those of the control chicks (Steel’s test, bicuculline, *t* = 0.86, n.s.; baclofen, *t* = 0.83, n.s.). The data of sham control chicks shown in Figure 2D are duplicated for comparison. n.s., not significant.

Injection of GABA-A receptor antagonists or GABA-B receptor agonists before the training made chicks imprintable on day 4 without T_3_ administration (Figure 2). To examine whether the effects of GABA-A receptor antagonists or GABA-B receptor agonists lasts for 4 days similar to the T_3_ effects in MP, we injected the GABA-A receptor antagonist bicuculline or the GABA-B receptor agonist baclofen on day 1, then trained and tested the chicks on day 4 (Figure 7A). The preference scores of these chicks were not different from those of the sham control chicks (Figure 7B). This result shows that the effects of either GABA-A receptor antagonists or GABA-B receptor agonists do not last for 4 days such as in MP. This indicates that GABA-B receptor signaling is necessary for acquiring the imprinting ability downstream of T_3_ but insufficient to induce MP. Most likely, GABA-B receptor signaling is only partly involved in T_3_ signaling, e.g., in neural transmission or intracellular molecular signaling, but not in T_3_-induced structural changes of neurons.

## Discussion

Chicks become imprintable to recognize their mothers and siblings at the appropriate time of the sensitive period. During that period, intracerebral T_3_ levels are critical to induce imprinting. Here, we showed using pharmacological approaches that the inhibitory neurotransmitter GABA contributes to the imprinting process via two types of receptors downstream of T_3_. Both ionotropic GABA-A and metabotropic GABA-B receptors play important roles at the start and the end of the imprinting-sensitive period. GABA binds to GABA-A and GABA-B receptors with similar affinities (Enna and McCarson, 2013). This suggests that the balance in GABA-A and GABA-B receptor expression can be a critical factor for mediating the intracellular signaling in the course of imprinting. This idea is consistent with the experiments using chicks injected with various receptor agonists and antagonists in the present paper. During the development, the role of GABA in imprinting is likely to shift as the quantitative balance of the two GABA receptors changes over time. As a consequence, GABA-B receptor signaling in the IMM facilitates imprinting behavior on day 1, while GABA-A receptor signaling suppresses imprinting on day 4. Considering that the GABA-B receptor is abundant on day 1, it may be involved in the start of the sensitive period. By contrast, the GABA-A receptor is abundant on day 5, suggesting that it may be involved in the termination of the sensitive period.

**FIGURE 8:**
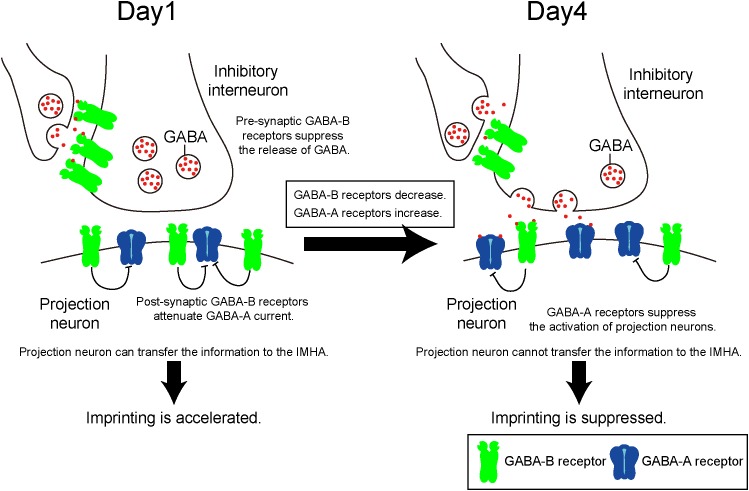
Quantitative balance of GABA-A and GABA-B receptors in the IMM determines the induction and termination of the sensitive period of imprinting. On day 1, GABA-B receptors are abundant compared to GABA-A receptors. Both pre- and post-synaptic GABA-B receptors may impair currents evoked by GABA-A receptor. Pre-synaptic GABA-B receptors suppress GABA release to projection neurons. Post-synaptic GABA-B receptors attenuate GABA-A receptor currents. From day 1 to day 4, the expression levels of GABA-B receptors decrease over time, while those of GABA-A receptors increase. Thus, GABA-A receptors become dominant and suppress neural activities of the projection neurons.

GABA-A receptors in mice function as post-synaptic Cl^-^ permeable heteropentameric ion channels that cause hyperpolarization (Jacob et al., 2008). The action of GABA-A receptors leads to a decreased depolarization induced by glutamatergic receptors, which is involved in the LTP that accompanies learning and memory. A previous paper has shown that imprinting in chicks is impaired after intraperitoneal injection of the GABA-A receptor modulator diazepam (Venault et al., 1986). In the present study, the GABA-A agonist muscimol suppressed imprinting behavior, and the expression levels of GABA-A receptors increased until day 5 when the sensitive period had already ended. These findings suggest that increased GABA-A receptor signaling suppresses IMM neuron activation, which impairs imprinting and terminates the sensitive period (Figure 8).

By contrast, the GABA-B receptor is a G protein–coupled receptor that is pre- and post-synaptically expressed (Gassmann and Bettler, 2012). Pre-synaptic GABA-B receptors reduce the Ca^2+^ influx through voltage-gated calcium channels, which inhibits neurotransmitter release. Accordingly, pre-synaptic GABA-B receptors reduce GABA release to post-synaptic GABA receptors (Deisz and Prince, 1989), suppressing the inhibitory action of post-synaptic GABA-A receptors. In addition, an electrophysiological experiment revealed that post-synaptic GABA-B receptors suppress the inhibitory action of GABA-A receptors of neurons in the mammalian amygdala and retina (Shen et al., 2017). Taken together, we hypothesize that during imprinting pre- and post-synaptic GABA-B receptors suppress the post-synaptic function of GABA-A receptors in different ways (Figure 8).

Thyroid hormone receptors are expressed in neurons of the IMM (Yamaguchi et al., 2012). T_3_ may mediate both GABA-A and GABA-B receptor signaling to facilitate imprinting in chicks. T_3_ reportedly reduces GABA-A receptor-evoked currents in the mammalian brain (Puia and Losi, 2011), suggesting that it may directly reduce the electrophysiological activity of GABA-A receptors. On the other hand, the phosphorylation level of nucleoside-diphosphate kinase 2 (NDPK2) is upregulated by T_3_ (Yamaguchi et al., 2016). NDPK2 is known to function downstream of the phosphoinositide 3-kinase (PI_3_K), which sends signals to open K^+^ channels (Srivastava et al., 2009), resulting in an enhanced post-synaptic GABA-B action. T_3_ may activate GABA-B receptors in projection neurons that were suppressed by GABA-A receptors.

Using immunostaining, we revealed that a significant number of larger neurons in the IMM are putative projection cells (Patel and Stewart, 1988; Tombol et al., 1988) that express both GABA-A and GABA-B receptors. This finding suggests that they are the projection neurons that receive GABA secreted from the pre-synapses of inhibitory interneurons. Projection neurons in the IMM project to the arcopallium and the IMHA (Bradley et al., 1985; Aoki et al., 2015). Our recent study shows that the neural connections from the IMM to the IMHA are critical for memory formation and recall in imprinting (Aoki et al., 2015). Information is likely to be transferred from the IMM to the IMHA neurons through the action of Wnt protein mediated by GABA receptors signaling, which is involved in the memory formation of imprinting (Yamaguchi et al., 2018). Additionally, mTOR whose activity is mediated by Wnt signaling (Ma et al., 2011) was recently demonstrated as an intracellular mediator downstream of T_3_ signaling in the course of imprinting (Batista et al., 2018). This suggests that GABA receptor signaling in the IMM mediates mTOR in IMHA neurons through Wnt protein signaling to induce imprinting.

Exogenous T_3_ administration induces imprintability even after the sensitive period has ended and extends the sensitive period for more than 1 week (Yamaguchi et al., 2012). In this study, GABA-B signaling was necessary for MP, while GABA-A signaling suppressed MP. However, chicks injected with either a GABA-B agonist or a GABA-A antagonist on day 1 could not be imprinted on day 4, indicating that both drugs fail to induce MP without the support of T_3_. Thus, GABA receptors are necessary but not sufficient for MP completion. GABA-A receptor antagonist and GABA-B receptor agonist may not induce in IMM neurons the structural changes that are necessary to accomplish MP.

## Conclusion

In the current study, we demonstrated that metabotropic GABA-B receptor signaling in the IMM is necessary for the acquisition of imprinting behavior, while ionotropic GABA-A receptor signaling suppresses imprinting. The quantitative balance between GABA-A and GABA-B receptors determines the duration of the imprinting-sensitive period. On day 1, when GABA-B receptors are abundant, chicks can be imprinted. By contrast, on day 4, when the GABA-A receptor expression level increases, chicks cannot be imprinted. Thereby, developmental changes in the GABA receptor balance determine the opening and the closing of the sensitive period.

## Author Contributions

NA designed the study, conducted experiments, and wrote the manuscript. KH contributed to interpretation of data and wrote the manuscript. SY, TF, CM, EF, and TM contributed to data collection and interpretation and critically reviewed the manuscript. All authors approved the final version of the manuscript, and agree to be accountable for all aspects of the work in ensuring that questions related to the accuracy or integrity of any part of the work are appropriately investigated and resolved.

## Conflict of Interest Statement

The authors declare that the research was conducted in the absence of any commercial or financial relationships that could be construed as a potential conflict of interest. The handling Editor and reviewer GV declared their involvement as co-editors in the Research Topic and confirm the absence of any other collaboration.
