# Supplementary material for: GABA-A and GABA-B Receptors in Filial Imprinting Linked With Opening and Closing of the Sensitive Period in Domestic Chicks (Gallus gallus domesticus)
Source: Front Physiol. 2018 Dec 19;9:1837. doi: 10.3389/fphys.2018.01837 (PMC6305906; doi:10.3389/fphys.2018.01837)
Supplement: Supplementary file 1 [file Data_Sheet_1.pdf]

Supplementary Table S1. List of GABA receptor chemicals used in each experiment

Experiment		Chemicals
Fig. 2B	GABA-B receptor agonist	Baclofen
	GABA-B receptor antagonist	CGP52432
Fig. 2D	GABA-B receptor agonist	Baclofen
	GABA-B receptor antagonist	CGP52432
Fig. 2E	GABA-A receptor agonist	Muscimol
	GABA-A receptor antagonist	Bicuculline
Fig. 2F	GABA-A receptor agonist	Muscimol
	GABA-A receptor antagonist	Bicuculline
	GABA-A receptor antagonist	Picrotoxin
	GABA-B receptor agonist	Baclofen
Fig. 3B	GABA-A receptor antagonist	Bicuculline
	GABA-B receptor agonist	Baclofen
Fig. 4B	GABA-A receptor agonist	Muscimol
	GABA-A receptor antagonist	Bicuculline
	GABA-B receptor agonist	Baclofen
	GABA-B receptor antagonist	CGP52432
Fig. 5B	GABA-A receptor agonist	Muscimol
	GABA-B receptor antagonist	CGP52432
Fig. 6B	GABA-A receptor agonist	Muscimol
	GABA-B receptor antagonist	CGP52432
Fig. 7B	GABA-A receptor antagonist	Bicuculline
	GABA-A receptor antagonist	Baclofen
