# Supplementary material for: GABA-A and GABA-B Receptors in Filial Imprinting Linked With Opening and Closing of the Sensitive Period in Domestic Chicks (Gallus gallus domesticus)
Source: Front Physiol. 2018 Dec 19;9:1837. doi: 10.3389/fphys.2018.01837 (PMC6305906; doi:10.3389/fphys.2018.01837)
Supplement: Supplementary file 2 [file Data_Sheet_2.pdf]

Supplementary Table S2. p values

Fig. 1A	Type of receptor	0.120
	Day	0.535
	Interaction	0.000
	Type of receptor on day1	0.000
	Type of receptor on day3	0.136
	Type of receptor on day5	0.003
	Days on GABA-A recepor	0.001
	Days on GABA-B recepor	0.002
Fig. 1B	GABA-B receptor	0.040
Fig. 1C	GABA-A receptor	0.034
Fig. 2B	Sham vs Baclofen	0.813
	Sham vs CGP52432	0.033
Fig. 2D	Sham vs Baclofen	0.015
	Sham vs Baclofen（i.v.）	0.004
	Sham vs CGP52432	0.290
Fig. 2E	Sham vs Muscimol	0.001
	Sham vs Bicuculline	0.916
	Sham vs GABA	0.207
Fig. 2F	Sham vs Muscimol	0.930
	Sham vs Bicuculline	0.012
	Sham vs Picrotoxin	0.001
	Sham vs Muscimol + Baclofen	0.999
Fig. 3B	Sham vs Low doses of bicuculline	0.448
	Sham vs Low doses of baclofen (i.v.)	0.397
	Sham vs Low doses of bicuculline + Low doses of baclofen (i.v.)	0.000
Fig. 4B	Sham vs T_3	0.000
	T_3 vs Muscimol	0.018
	T_3 vs Bicuculline	0.255
	T_3 vs Baclofen	0.856
	T_3 vs CGP52432	0.043
Fig. 5B	T_3 vs Muscimol	0.046
	T_3 vs CGP52432	0.016
Fig. 6B	T_3 vs Muscimol	0.006
	T_3 vs CGP52432	0.047
Fig. 7B	Sham vs Bicuculline	0.616
	Sham vs Baclofen	0.640
