# Supplementary material for: GABA-A and GABA-B Receptors in Filial Imprinting Linked With Opening and Closing of the Sensitive Period in Domestic Chicks (Gallus gallus domesticus)
Source: Front Physiol. 2018 Dec 19;9:1837. doi: 10.3389/fphys.2018.01837 (PMC6305906; doi:10.3389/fphys.2018.01837)
Supplement: Supplementary file 3 [file Data_Sheet_3.pdf]

Supplementary Table S3. Effect sizes

Fig. 1A	η^2	Type of receptor	0.060
		Day	0.030
		Interaction	0.687
Fig. 1B	*d*	GABA-B receptor	0.923
Fig. 1C	*d*	GABA-A receptor	0.890
Fig. 2B	*r*	Sham vs Baclofen	0.117
		Sham vs CGP52432	0.478
Fig. 2D	*r*	Sham vs Baclofen	0.521
		Sham vs Baclofen（i.v.）	0.605
		Sham vs CGP52432	0.293
Fig. 2E	*r*	Sham vs Muscimol	0.720
		Sham vs Bicuculline	0.117
		Sham vs GABA	0.163
Fig. 2F	*r*	Sham vs Muscimol	0.128
		Sham vs Bicuculline	0.551
		Sham vs Picrotoxin	0.649
		Sham vs Muscimol + Baclofen	0.035
Fig. 3B	*r*	Sham vs Low doses of bicuculline	0.209
		Sham vs Low doses of baclofen (i.v.)	0.231
		Sham vs Low doses of bicuculline + Low doses of baclofen (i.v.)	0.728
Fig. 4B	*r*	Sham vs T_3	0.730
		T_3 vs Muscimol	0.500
		T_3 vs Bicuculline	0.278
		T_3 vs Baclofen	0.210
		T_3 vs CGP52432	0.469
Fig. 5B	*r*	T_3 vs Muscimol	0.544
		T_3 vs CGP52432	0.562
Fig. 6B	*r*	T_3 vs Muscimol	0.562
		T_3 vs CGP52432	0.410
Fig. 7B	*r*	Sham vs Bicuculline	0.163
		Sham vs Baclofen	0.160
