# Supplementary figures and images for: GABA-A and GABA-B Receptors in Filial Imprinting Linked With Opening and Closing of the Sensitive Period in Domestic Chicks (Gallus gallus domesticus)
Source: Front Physiol. 2018 Dec 19;9:1837. doi: 10.3389/fphys.2018.01837 (PMC6305906; doi:10.3389/fphys.2018.01837)

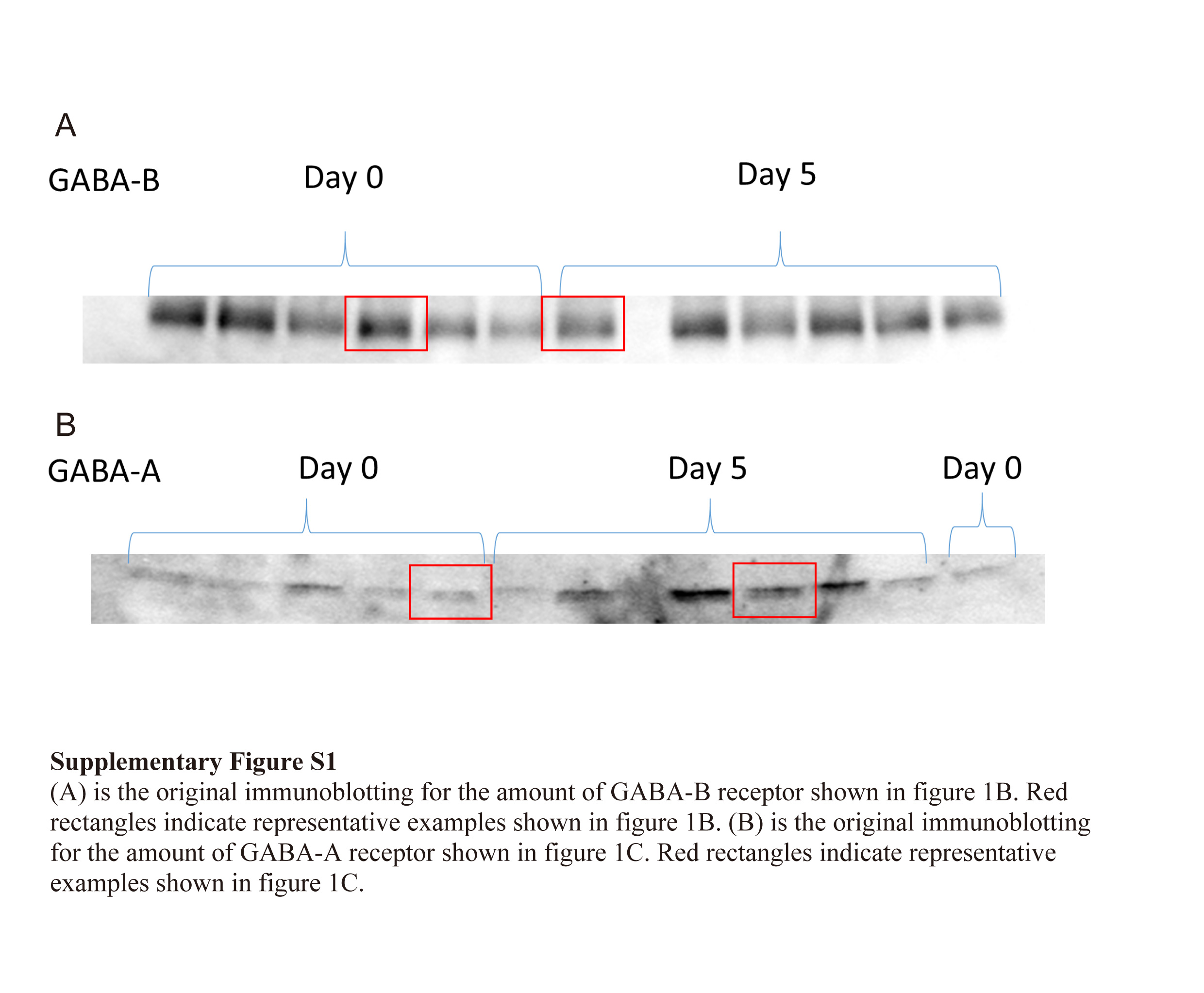

Supplement: Supplementary file 4 [file Image_1.jpg]
